# A Proven Case of Cutaneous *Rhizopus* Infection Presenting with Severe Limb Pain Very Soon after Induction Treatment in a Patient with Acute Lymphoblastic Leukemia

**DOI:** 10.1155/2015/285360

**Published:** 2015-02-15

**Authors:** Mehmet Sezgin Pepeler, Kadir Acar, Özlem Güzel Tunçcan, Ömer Uluoğlu, Ayşe Kalkancı, Hakan Atalar, Koray Kılıç, Gülsan Türköz Sucak

**Affiliations:** ^1^Department of Hematology, Gazi University Faculty of Medicine, Ankara, Turkey; ^2^Department of Infectious Disease, Gazi University Faculty of Medicine, Ankara, Turkey; ^3^Department of Pathology, Gazi University Faculty of Medicine, Ankara, Turkey; ^4^Department of Microbiology, Gazi University Faculty of Medicine, Ankara, Turkey; ^5^Department of Orthopedics, Gazi University Faculty of Medicine, Ankara, Turkey; ^6^Department of Radiology, Gazi University Faculty of Medicine, Ankara, Turkey

## Abstract

*Objective and Importance*. Invasive mucormycosis may complicate the course of patients with hematologic malignancies and has a very high mortality rate. Early diagnosis and aggressive approach combined with surgical and medical treatment have paramount importance for cure. *Clinical Presentation*. We report here a case of a patient with acute lymphoblastic leukemia presenting with a subcutaneous mass lesion which was sampled by an ultrasound guided needle biopsy. The pathology showed microorganisms with aseptate hyphae with wide, irregular walls and more or less branching with highly vertical angles which suggested a mold infection. The specimen was also cultured where *Rhizopus* spp. grew. *Conclusion*. Posaconazole 200 mg QID was commenced. She recovered from neutropenia and pain on day 20 of treatment. After 4 courses of hyper-CVAD chemotherapy, the remaining soft tissue mass was removed surgically and she underwent allogeneic HSCT from a full matched sibling donor under secondary prophylaxis.

## 1. Introduction

Invasive fungal infections are major causes of morbidity and mortality in immunosuppressed patients. Invasive mucormycosis (IMM) is the third most frequent invasive fungal infection after aspergillosis and candidiasis in patients with acute leukemia [1]. The incidence has risen significantly in the past decades with the rapidly growing number of highly immunosuppressive treatment modalities such as stem cell and solid organ transplantation and the use of broad spectrum antimicrobial and antifungal agents. The established risk factors are immunosuppression, diabetes mellitus, iron overload, deferoxamine treatment, and graft versus host disease [1]. Here, we present a patient with none of the above risk factors; a young agricultural worker is on the eighth day of leukemia induction chemotherapy. She was neutropenic, though not for a long time, and was not on broad spectrum antibiotics and she was not diabetic. We believe that her occupation was the underlying cause and the port of entry for the pathogen was a possibly an unnoticed point of skin dehiscence which occurred before the diagnosis of leukemia.

## 2. Case Report

A 27-year-old female, who worked as an agricultural laborer, was referred to our hospital with complaints of fatigue, malaise, anorexia, and epistaxis. Her physical examination revealed pallor and eczematous plaques in her chest and the proximal part of her right arm. She also had splenomegaly of 6 cm below the costal margin. Laboratory investigation revealed a hemoglobin level of 7,3 g/dL, white blood cells of 4,0 × 10^9^/L, and a platelet count of 23 × 10^9^/L. Her peripheral blood smear revealed lymphoblasts which came out to be TdT, CD34, CD19, CD10, CD22, and HLA-DR positive with flow cytometry. Bone marrow analysis with cytogenetic and microscopy confirmed the diagnosis of Acute Lymphoblastic Leukemia. The eczematous plaques were considered as contact dermatitis and topical methylprednisolone aceponate was commenced. The lesions recovered rapidly and completely short after topical treatment.

**Figure 1:**
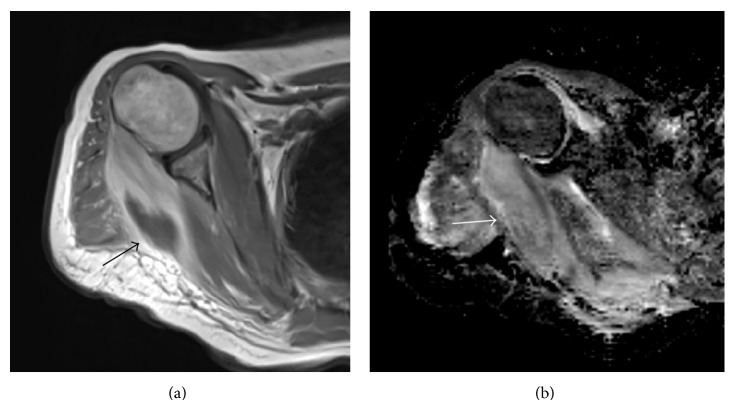
(a)* Axial MRI* image of the* Rhizopus* abscess. Contrast enhanced T1W image shows thickening of the teres minor and major muscles with heterogeneous enhancement and hypointense central area of necrosis suggestive of abscess (black arrow). (b) High signal in the ADC ap of DW sequence (*b* = 600 s/mm^2^ in the diffusion (white arrow) (MRI: magnetic resonance imaging; T1W: t1 weighted; ADC: apparent diffusion coefficient; DW: diffusion-weighted)).

**Figure 2:**
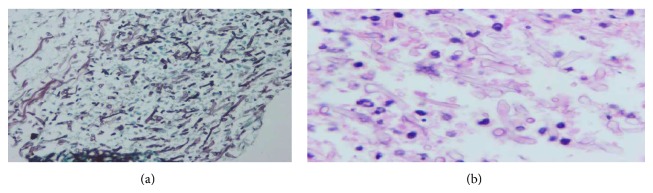
(a) Grocott's methenamine silver stained histopathological specimen of necrotic cutaneous tissue demonstrating aseptate hyphae (×40). (b) Hematoxylin and eosin (H&E) stained histopathological specimen of necrotic cutaneous tissue demonstrating aseptate hyphae (×100).

**Figure 3:**
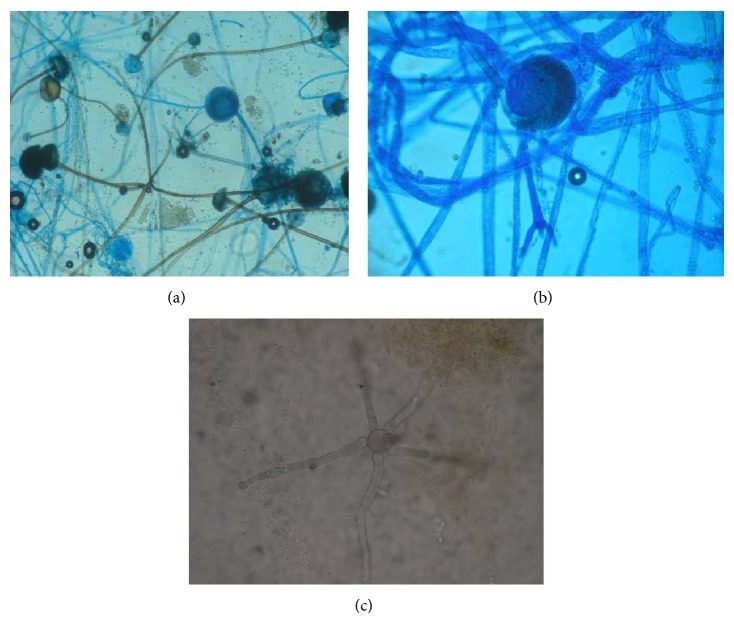
(a) Structural features of* Rhizopus* species. The sporangiophores (a stalk that arises from the vegetative hypha) and sporangia (asexsual spore-forming structures) are visible as they are rising from stolons opposite to rhizoids. (Lactophenol cotton blue preparation from culture, 40x magnification.) (b) A sporangiophore filled by sporangiospores. (Lactophenol cotton blue preparation from culture, 40x magnification.) (c) KOH preperation of tissue sample shows coenocytic (nonseptated) hypha (40x magnification).

Hyper-CVAD (cyclophosphamide, vincristine, doxorubicin, dexamethasone, l-asparaginase, and intrathecal therapy) protocol was started immediately after diagnosis. She developed febrile neutropenia 8 days after admission and piperacillin tazobactam was started. Teicoplanin was added to her treatment as coagulase-negative* Staphylococcus* species grew in her blood cultures. Repeated blood cultures due to persistent fever revealed* Enterococcus* sp. Teicoplanin was replaced with daptomycin. High resolution chest tomography (HRCT) revealed nonspecific nodules under 1 centimeter. Serum galactomannan antigen was negative. After 10 days of persistent fever with an absolute neutrophil count of 20/*μ*L, she developed a very severe pain in her right arm radiating to her shoulder suggesting herpes zoster infection due to dermatome distribution. However, there were no vesicles in her physical examination. A very tender point in the upper lateral scapular region with a vague mass lesion was found. Magnetic resonance imaging (MRI) of the right shoulder region showed a focal cystic necrotic lesion between the teres minor and teres major muscle groups (Figures 1(a) and 1(b)). An ultrasound guided fine needle biopsy was performed. The microscopic examination of the biopsy sections stained with hematoxylin and eosin Grocott's methenamine silver showed aseptate hyphae with wide, irregular walls and more or less branching with highly vertical angles (Figures 2(a) and 2(b)).* Rhizopus* species grew in the culture (Figures 3(a), 3(b), and 3(c)). Posaconazole 200 mg QID was started. She defervesced a few days after posaconazole and recovered from her neutropenia on day 20 of treatment. The pain and the mass in her scapular region recovered as well. She completed 4 courses of hyper-CVAD regimen. The remaining mass lesion, smaller in size was removed by our orthopedic surgeons. She received an allogeneic HSCT from her full matched sister with secondary antifungal prophylaxis with amphotericin B. She is currently 4-month status post an uneventful allogeneic HSCT and is off of antifungal treatment.

## 3. Discussion

Invasive mucormycosis is the third most common fungal agent which complicates the course of patients with hematological malignancies, with very high mortality rates [2]. The incidence of IMM has been rising in the past few decades in immunocompromised patients though it can also be seen in immunocompetent people unlike other fungal pathogens causing invasive fungal infections. Growing numbers of stem cell and solid organ transplants and the increased use of antimicrobial and antimold agent voriconazole has been accounted for this increased incidence. Inhalation, oral ingestion, and inoculation through cutaneous exposure are the major routes of entry.* Rhizopus* species is the most common species among Mucoromycotina (formerly Zygomycetes) infections particularly the cutaneous forms, comprising almost half of IMM cases [1].

Among the five distinct forms including rhino cerebral, pulmonary, cutaneous, gastrointestinal, and disseminated forms, pulmonary and rhino cerebral forms are the leading forms in leukemic patients. Contrary to general opinion, cutaneous form is also seen in leukemia patients though with a relatively less frequency and less mortality. This form is observed in muscle and fascia and occasionally causes bone involvement [3].

The presented case has some instructive features which we believe should be addressed. First of all, invasive fungal infection was not a part of our differential diagnosis at the beginning due to relatively short duration of neutropenia and absence of long term antimicrobial treatment. However, after the diagnosis of proven invasive fungal infection, we retrospectively considered that the erythematous plaques found just after admission could have been a cutaneous fungal infection and should have been sampled for fungus. There are similarly very early cases of IMM with a very short period of neutropenia [4]. Zygomycetes are found in soil in ample amounts.

Thus, the occupation of the patient as an active agricultural worker suggests that an unnoticed skin dehiscence could have been the port of entry for the fungal agent.

Though liposomal amphotericin B was the antifungal agent chosen for secondary antifungal prophylaxis during her transplant course, the initial febrile episode was successfully controlled by posaconazole. There are conflicting reports regarding the efficacy of posaconazole in IMM [5–7]. There are reports questioning the efficacy of posaconazole against mucormycosis as some transplant recipients developed IMM under posaconazole treatment [8]. Van Burik et al. reported successful treatment of IMM with posaconazole in 60% of their patients [9]. There are also cases resistant to amphotericin B who responded to posaconazole [6]. An in vitro study from Turkey has demonstrated more potent activity of posaconazole against* Rhizopus oryzae* compared to voriconazole, itraconazole, and amphotericin B [10]. These conflicting results could be easily explained by the erratic absorption of posaconazole and the selection of resistant strains under posaconazole prophylaxis. Our patient was antifungal naive and tolerated and responded to posaconazole very well, suggesting that the drug reached efficient serum levels and explaining her excellent outcome. It should also be noted that the histopathology of the final mass lesion removed just before transplantation showed no signs of infection which indicates that the infection was eradicated with posaconazole. The deeper extension of cutaneous form of the infection in the presented case is not surprising as almost half of the cutaneous infections in the largest series reported had been either extended to adjacent soft tissues or disseminated [6].

The presented case suggests that IMM may not necessarily occur in patients with prolonged neutropenia and who are under broad spectrum antibacterial or antifungal prophylaxis. Suspicion index should be kept high particularly in patients with occupational risk factors and subtle signs like skin rash. Although posaconazole was safe and effective in the presented patient, it is not possible to draw a solid conclusion.

Early diagnosis and aggressive combined approach with surgical and medical treatment have paramount importance for cure.
